# Utilization of agro-industrial waste for β-galactosidase production under solid state fermentation using halotolerant *Aspergillus tubingensis* GR1 isolate

**DOI:** 10.1007/s13205-014-0236-7

**Published:** 2015-01-15

**Authors:** Gopal G. Raol, B. V. Raol, Vimal S. Prajapati, Nirav H. Bhavsar

**Affiliations:** 1 Department of Microbiology, Shri A.N. Patel P.G. Institute, Sardar Patel University, Anand, Gujarat India; 2 Department of Microbiology, Shri P. H. G. Muni. Arts and Science College, Gujarat University, Kalol, Gujarat India; 3 B.R.D. School of Biosciences, Sardar Patel University, Vallabh Vidhyanagar, Gujarat India

**Keywords:** Agro-industrial waste, Halotolerant, Solar saltern, *Aspergillus tubingensis*, Deproteinized acid cheese whey, Response surface methodology

## Abstract

A halotolerant fungal isolate *Aspergillus tubingensis* GR1 was isolated from the man-made solar saltern located at Khambhat, Gujarat, India, and identified using 28S rDNA partial genome sequencing. This isolate was studied for β-galactosidase production under solid state fermentation using wheat bran and deproteinized acid cheese whey. The influence of various agro-industrial wastes, nitrogen source and other growth conditions on β-galactosidase production was investigated using ‘one-factor-at-a-time’ approach. Among various variables screened along with wheat bran and deproteinized acid cheese whey as major growth substrate, corn steep liquor and MgSO_4_ were found to be most significant. The optimum concentrations of these significant parameters were determined employing the response surface central composite design, revealing corn steep liquor concentration (2 mL) and magnesium sulphate (50 mg) per 5 g of wheat bran and 20 mL of deproteinized acid cheese whey for highest enzyme production (15,936 U/gds). These results suggest the feasibility of industrial large-scale production of β-galactosidase known to be valuable in whey hydrolysis and removal of galactosyl residue from polysaccharide.

## Introduction

India being an agriculture-dominant country produces more than 500 million tons of crop residues annually (MNRE 2009). These residues are used as animal feed, for thatching of homes, and as a source of domestic and industrial fuel. A large portion of unused crop residues are burnt in the fields primarily to clear the left-over straw and stubbles after the harvest. Non-availability of labour, high cost of residue removal from the field and increasing use of combines in harvesting the crops are main reasons behind burning of crop residues in the fields. Burning of crop residues causes environmental pollution, is hazardous to human health, produces greenhouse gases causing global warming and results in loss of plant nutrients like nitrogen, phosphorus, potassium and sulfur. India is the leading producer of milk in the world; about 2 and 1.5 million tons of channa and paneer (cottage cheese), respectively, are produced annually and during their production, about 75–85 % of the volume of milk is removed as whey (Aneja et al. 2002). The disposal of whey remains a serious problem for dairy industry, especially in developing countries where a relatively insignificant part of whey is used for production of whey protein concentrates or permeates. A major part of whey is disposed into the water streams, causing serious water pollution problems arising from high biological oxygen demand (BOD) mainly because of the presence of 5 % dissolved solids, of which lactose is the main constituent (Marwaha and Kennedy 1988). Therefore, appropriate management of such generated agro-industrial waste assumes a great significance. However, such wastes usually have a composition rich in sugars, minerals and proteins, and hence they should not be considered “wastes” but raw materials for other industrial processes. A number of applications for utilization of such agro-industrial wastes have been developed to overcome the problem of its disposal. In order to be economically attractive, these applications must go beyond the traditional use as additives for animal feedstock. One alternative is the use of whey and other agro-industrial wastes as the basic medium for various fermentation processes including the production of industrially important enzymes, ethanol, methane, yeast protein, xanthan gum and various organic acids (Siso 1996; Pandey et al. 1999).

β-d-Galactosidase (EC 3.2.1.23, β-d galactoside, galactohydrolase, lactase) catalyzed the non-reducing β-d-galactosyl residues from polysaccharides. They are widely distributed in plants, microorganisms and animals (Kumari et al. 2011; Vermaa et al. 2012). Among these, microbial sources offer several advantages, such as easy handling and high production yields. Commercially available β-galactosidase is obtained from microorganisms of different genera in particular from *Kluyveromyces*, *Candida*, *Aspergillus*, *Bacillus* spp. and *E. coli* (Panesar et al. 2006; Thigiel and Deak 1989; Pinheiro et al. 2003). This enzyme has many industrial and medicinal applications like cleavage of blood group A and B glycotopes, biosensors for lactose determination and enzymatic hydrolysis of lactose (Asraf Shaikh and Gunasekaran 2010). Especially the enzymatic hydrolysis of lactose has many advantages in food industry. Lactose hydrolyzed products decrease the lactose intolerance problem (Mlichova and Rosenberg 2006).

Conventional practice of single factor optimization by maintaining other factors at an unspecified constant level does not depict the combined effect of all the factors involved. The single variable optimization methods are not only tedious, but can also lead to misinterpretation of results, especially because the interaction between different factors is overlooked. However, the ‘one-variable-at-a-time’ approach may be useful for estimating suitable operational intervals for important inhibitory or stimulation variables prior to conducting response surface studies. Statistical methodologies can be applied in biotechnological processes to define the main effects and interactions of the factors that play fundamental roles in the fermentations. Response surface methodology (RSM) is a powerful technique for testing multiple process variables because fewer experimental trials are needed compared to the study of one variable at a time and the interactions between variables can be identified and quantified by such technique (Prajapati et al. 2013; Vaidya et al. 2003). *Aspergillus* spp. also produces β-galactosidase by fermentation on different carbon sources (Maksimainena et al. 2013; Awan et al. 2010; Nizamuddin et al. 2008; Oberoi et al. 2008; Gindy et al. 2009) but a detailed statistical medium optimization studies using agro-industrial wastes are not available for producing β-galactosidase using halotolerant *Aspergillus* spp.

The aim of the present study is to utilize the various agro-industrial wastes for production of β-galactosidase under SSF. Response surface methodology has been employed for the level optimization of screened significant components for enhanced β-galactosidase production from halotolerant *A. tubingensis* GR1.

## Materials and methods

### Chemicals

All chemicals were used of analytical grade and commercially available. *O*-Nitrophenyl- β-d-galactopyranoside (ONPG), and X-Gal were purchased from HiMedia (Mumbai, Maharashtra, India). Wheat bran was procured from Lisa Roller Mill Industries (Gandhinagar, Gujarat, India). Corn steep liquor was obtained from Maize Products Industries (Ahmedabad, Gujarat, India). Cheddar cheese whey was procured from Vidya Dairy Cheese Processing Plant, Khatraj, Anand, India. Other agricultural wastes like cabbage leaves, orange peel, rice polishing waste, sugarcane bagasse and groundnut deoiled seed cake were procured from local farm and market, Anand.

### Strain isolation and identification

Halotolerant fungus was isolated from the sediment soil samples collected from the man-made solar saltern located at Khambhat, Bay of Cambay, Gujarat, India. The fungus isolate was checked for β-galactosidase activity qualitatively by growing on Minimal Nutrient Salt medium containing 0.04 g % (w/v) X-Gal. The culture was maintained at 4 °C on potato dextrose agar supplemented with 70 g/L crude NaCl (local market available edible salt). The selected fungal isolate was identified using FAST Microseq^®^ D2 LSU rDNA fungal identification kit (Applied Biosystems, Foster city, CA, USA). DNA extraction was carried out using Prepman™ ultra sample preparation reagent and D2 LSU rRNA gene was amplified and cycle sequencing was carried out as per the kit instructions. Amplification was carried out in a thermal cycler (9800, Applied Biosystems, Foster city, CA, USA) with reaction profile: initial denaturation at 95 °C for 10 s followed by 35 cycles of denaturation 95 °C for 30 s, annealing at 64 °C for 15 s, extension at 72 °C for 1 min and finally extension at 72 °C for 5 min. The purified PCR product was sequenced and the phylogenic relationship of the isolate was determined by comparing the sequence data with the existing sequences available through the gene bank database of the National Center for Biotechnology Information (NCBI, Bethesda, MD, USA).

### β-galactosidase production under SSF

β-galactosidase production was carried out in 250 mL Erlenmeyer flasks containing 5 g washed, dried and finely powdered (2–4 mm particle size) agro-residues as substrates, and moistened with two different moistening mediums, deproteinized acid cheese whey and distilled water. Deproteinized acid cheese whey and substrate were sterilized separately at 121 °C at 15 lbs for 15 min, mixed at the time of inoculation with 1 mL inoculum (1 × 10^6^ Spores/mL), and incubated at 28 °C under static conditions. The flasks were shaken intermittently (twice a day) to ensure homogeneous mixing of contents. The enzyme was extracted from each flask at regular time intervals and crude enzyme was used for further analysis.

Inoculum was prepared from 10 days grown culture on potato dextrose agar slants containing 7 % NaCl. Spore suspension was prepared by adding sterile distilled water containing 0.01 % Triton X-100 to each slant and was brushed lightly with a sterile wire loop. Spore count was done using Neubauer counting chamber.

### Enzyme extraction and assay

Enzyme was extracted using 30 mL 75 mM sodium-citrate buffer (pH 4.0) and filtered through wet muslin cloth by thorough squeezing. The extract was centrifuged at 7,500 *g* for 15 min. Clear supernatant after centrifugation was used as an enzyme source for determining β-galactosidase activity. Enzyme activity was assayed in the reaction mixture containing 0.9 mL of sodium-citrate buffer (75 mM, pH 4.0) and 0.1 mL of enzyme source. Add 1.0 mL of 2 mM *O*-nitrophenyl-β-d-galactopyranoside (ONPG) into this mixture followed by incubation at 50 °C for 5 min. The reaction was stopped by addition of 3.0 mL 0.1 N NaOH. Released *O*-nitrophenol was determined spectrophotometrically at 420 nm using calibration curve prepared with *O*-nitrophenol under the same conditions (Cruz et al. 1999). One unit (U) of β-galactosidase activity was defined as the enzyme librating one µM *O*-nitrophenol (molar extinction coefficient of ONP is *ε*_*m*_ = 4,166 L mol^−1 ^cm^−1^) per 1 min under the standard assay conditions.

### Optimization of process parameters

The various process parameters such as agro-residues, particle size of residue, substrate to moisture ratio, incubation time, temperature, inoculum size, nitrogen sources and trace elements which influence the enzyme production under SSF were optimized over a wide range. The strategy was adopted for standardization of fermentation parameters to evaluate the effect of an individual parameter and to incorporate it at standardized level before standardizing the next parameter.

β-galactosidase production was carried out under SSF as described earlier using 5 g washed, dried and finely powdered (2–4 mm particle size) agro-residues viz. wheat bran, rice polishing waste, cabbage leaves, orange peel, sugarcane bagasse and groundnut deoiled seed cake. The effect of two different moistening mediums, viz. deproteinized acid cheese whey and distilled water on enzyme production was studied at 1:3 ratio of substrate to moistening medium. Effect of different particle size of wheat bran was studied by grinding the wheat bran to 4,000 > Particle Size (PS) > 2,000, 2,000 > PS > 1,000, 1,000 > PS > 500, 500 > PS > 250 and 250 > PS > 150 (µm) and moistened with 15 mL deproteinized acid cheese whey (DACW). The effect of moisture level on β-galactosidase production was studied by varying the ratio of substrate (wheat bran 250 > PS > 150) to moistening medium (DACW) from 1:2 to 1:5 (w/v). The effect of incubation period on enzyme activity was examined at different time intervals of 4, 5, 6, 7, 8, and 9 days respectively, at ambient temperature (28 ± 3 °C). The effect of temperature on enzyme production was examined at 24, 28, 32, 36 and 40 °C using above optimized media content and activity was measured on eighth day of fermentation.

The effect of inoculum size on the enzyme production was determined by adding the spore suspension in order of 1 × 10^4^, 1 × 10^5^, 1 × 10^6^ and 1 × 10^7^ spores/mL to production flasks. Fermentation was carried out at 28 °C temperature for 8 days. The effect of supplementation of additional nitrogen sources to wheat bran and DACW was examined using nitrogen sources consisting of peptone, soya bean meal, corn steep liquor (CSL), ammonium sulphate, sodium nitrate and urea at the rate of 1 g per 5 g of wheat bran (1 mL in case of corn steep liquor). The effect of trace element on the β-galactosidase production was studied by supplementing 10 mg of MgSO_4_, FeSO_4_, ZnSO_4_, NaCl and CaCl_2_ into each separate Erlenmeyer flask containing, 5 g wheat bran, 1 mL CSL and 20 mL DACW. Fermentation was carried out at 28 °C temperature for 8 days.

### Optimization of the medium components for increased β-galactosidase production

The most important parameters, which affect the efficiency of β-galactosidase production, were found to be magnesium sulphate and corn steep liquor. In order to study the individual and combined effect of these parameters, statistically designed experiments were performed.

#### Central composite design (CCD)

The levels of the significant parameters as revealed from ‘one-factor-at-a-time’ approach experiment and their interaction effects which influence the β-galactosidase production were analyzed and optimized by response surface central composite design (CCD). RSM is useful for small number of variables (up to five) but is impractical for large number of variables, due to high number of experimental runs required. The level of the two major components MgSO_4_ and corn steep liquor were optimized, keeping other parameter namely wheat bran, moistening medium, temperature, time interval, and inoculum size at a constant level.

According to the design, the total number of treatment combinations is 2^*k*^ + 2*k* + no, where *k* is the number of independent variables and no is the number of repetition of experiments at the central point. Each factor in the design was studied at five different levels (−α, −1, 0, +1, +α) as shown in Table 1. This experimental design comprises a two level fractional factorial points (−1 and +1), central point (0) and axial or star points encoded as −α and +α. All variables were set at a central coded value of zero. The minimum and maximum ranges of variables were determined on the basis of our previous experiments. The full experimental plan with respect to their values in actual and coded form is listed in Table 2. β-galactosidase (U/gds) was measured in triplicate in 14 different experimental runs. β-galactosidase production was analyzed using a second order polynomial equation and the data were fitted into the equation by multiple regression procedure. The model equation for analysis is given as:

**Table 1:** Experimental range and levels of the independent variables of selected components used for response surface central composite design

Variable	Components	Range	Levels of variable studied				
			−α	−1	0	+1	+α
X1	MgSO_4_ (w/w)	1.0–10.0	−0.863	1.0	5.5	10.0	11.86
X2	Corn steep liquor (v/w)	0.02–0.4	−0.0587	0.02	0.21	0.4	0.478

**Table 2:** Full experimental central composite design with coded and actual level of variables and the response function

Run no.	A: MgSO_4_ (w/w)		B: Corn steep liquor (v/w)		β-galactosidase production (U/gds)	
	Actual level	Coded level	Actual level	Coded level	Observed	Predicted
1	1	−1	0.02	−1	5,040	5979.07
2	10	+1	0.02	−1	4,464	4778.47
3	1	−1	0.4	+1	12,624	12803.78
4	10	+1	0.4	+1	15,936	15491.17
5	−0.863	−α	0.21	0	10,480	9791.21
6	11.86	+α	0.21	0	10,648	10842.54
7	5.5	0	−0.0587	−α	3,793	3008.96
8	5.5	0	0.478	+α	15,120	15409.78
9	5.5	0	0.21	0	11,520	11,259
10	5.5	0	0.21	0	11,664	11,259
11	5.5	0	0.21	0	11,568	11,259
12	5.5	0	0.21	0	10,472	11,259
13	5.5	0	0.21	0	10,906	11,259
14	5.5	0	0.21	0	11,424	11,259

1 $$Y \, = \, \beta_{0} + \, \Sigma \, \beta iXi \, + \, \Sigma \, \beta iiXi^{2} + \Sigma \, \beta ijXiXj$$

where,* β*_0_ *, βi, βii* and *βij* represent the constant, linear, quadratic effect of *Xi* and interaction effect between *Xi* and *Xj*, respectively for the production of β-galactosidase. Later, validation experiment was performed and maximum production of β-galactosidase was confirmed using the optimum values for variables predicted by response optimization.

#### Software and data analysis

The results of the experimental design were analyzed and interpreted using Design Expert Version 8.0 (Stat-Ease Inc., Minneapolis, Minnesota, USA) statistical software.

## Results and discussion

### Identification of the fungal isolate

**Fig. 1:**
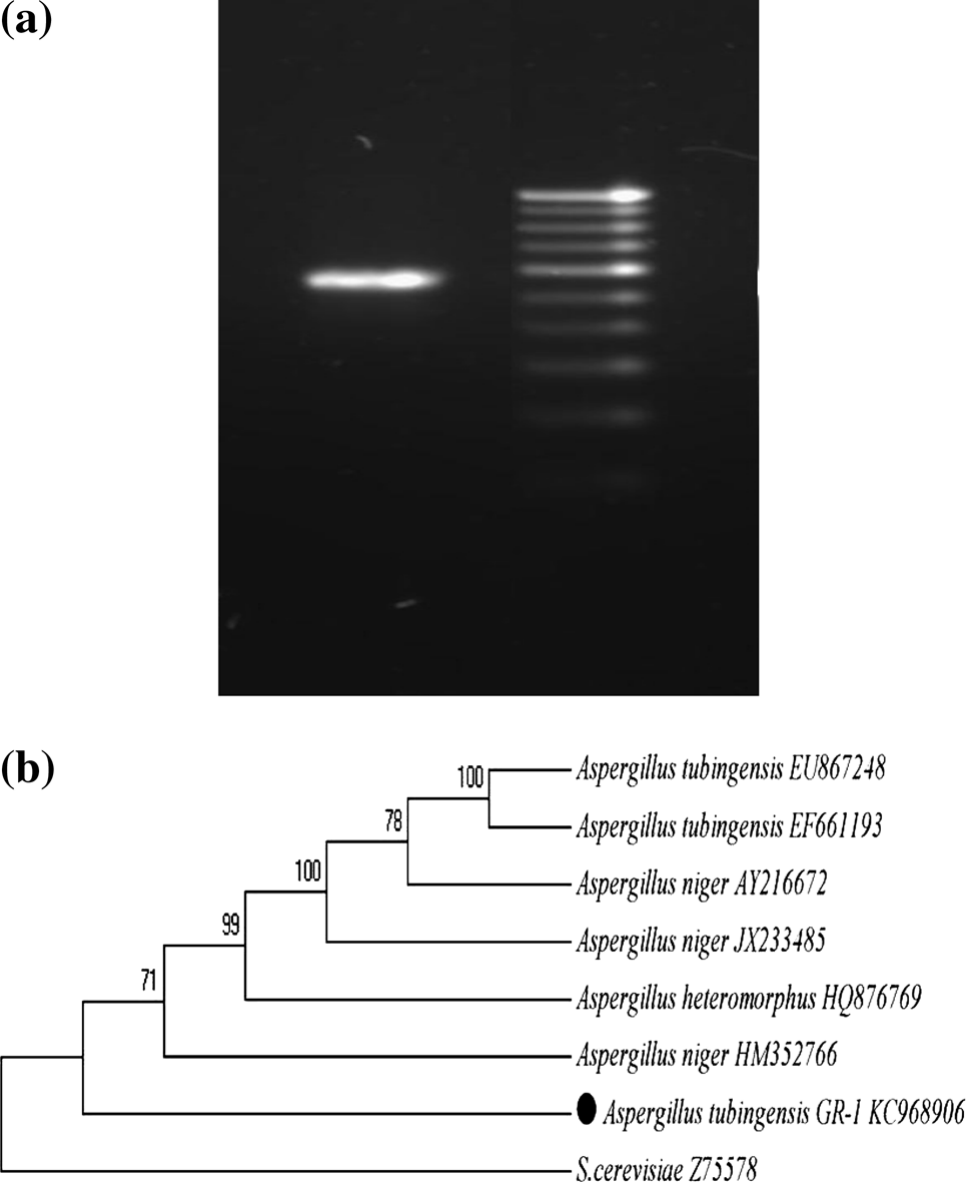
**a** 618 bp PCR product with DNA marker. **b** Phylogenetic relationship on the basis of homology index for a halotolerant fungal isolate *A. tubingensis* GR1

A 618 bp size 28S rDNA sequence of the isolate was obtained through PCR amplification (Fig. 1a). The sequence had 97 % homology with *A. tubingensis* showing 928 maximum score having 0.00 e-value. The sequence was deposited in the NCBI gene bank bearing the Accession no. KC968906. The evolutionary history was inferred using the Neighbor-Joining method (Saitou and Nei 1987). The bootstrap consensus tree inferred from 1,000 replicates is taken to represent the evolutionary history of the taxa analyzed. Branches corresponding to partitions reproduced in less than 50 % bootstrap replicates are collapsed. The percentage of replicate trees in which the associated taxa clustered together in the bootstrap test (1,000 replicates) are shown next to the branches (Felsenstein 1985). The analysis involved 8 nucleotide sequences and the ambiguous positions were removed for each sequence pair. There were a total of 1,814 positions in the final dataset. The phylogenic tree was drawn using bioinformatics software MEGA 5.05 (Fig. 1b) (Raol et al. 2014).

### β-galactosidase production using different agro-industrial wastes under SSF by *A. tubingensis* GR1

**Fig. 2:**
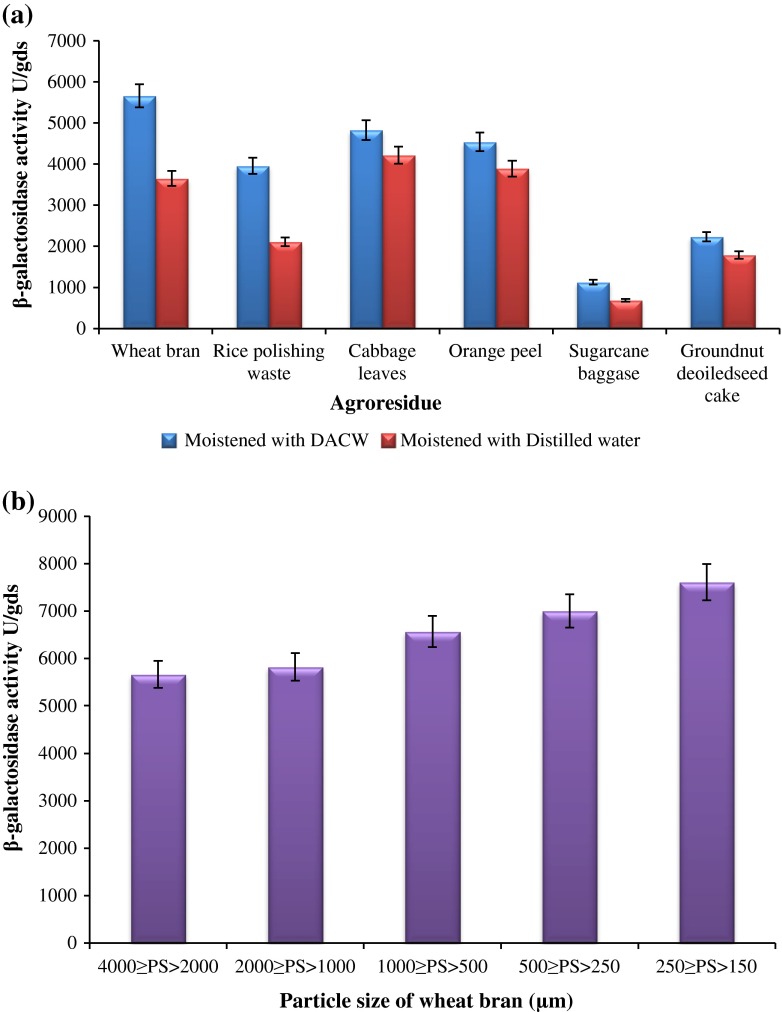
**a** Effect of different agro-residues on β-galactosidase production. **b** Effect of different particle size of wheat bran on β-galactosidase production

β-galactosidase production was carried out initially using various agro-industrial wastes viz. wheat bran, rice polishing waste, cabbage leaves, orange peel, sugarcane baggase and deoiled groundnut seed cake as substrate and moistened with two different moistening medium i.e. distilled water and deproteinized acid cheese whey at 1:3 ratio of substrate to moistening medium under SSF at 28 ± 3 °C temperature (Fig. 2a). Maximum β-galactosidase production (5661.4 ± 20.9 U/gds) was observed after 7 days of incubation when wheat bran was moistened with deproteinized cheese whey whereas, distilled water as moistening medium yields low enzyme production (3,648 ± 24 U/gds). However, when cabbage leaves were moistened with distilled water gave maximum enzyme production (4216.3 ± 14 U/gds) compared to wheat bran (3,648 ± 24 U/gds). Cabbage leaves yielded enzyme activity up to 4824.64 ± 15.3 U/gds while the other substrates did not favor significant enzyme production. Wheat bran has been reported to induce the expression of galactosidases in various microbial species due to its high hemicelluloses content, total sugar available, presence of suitable nutrient and favorable degradability (Miradamadi et al. 1997; de Vries and Visser 2001; Awan et al. 2010). In the present study, we found that wheat bran along with deproteinized cheese whey induces elevated level of β-galactosidase compared to wheat bran alone. Literature revealed that particle size of the substrate has showed prominent effect on the enzyme production. In present investigation, separate experiment was carried out considering the different particle size of wheat bran ranging 4,000 > PS > 2,000, 2,000 > PS > 1,000, 1,000 > PS > 500, 500 > PS > 250 and 250 > PS > 150 (µm). The results were depicted in Fig. 2b, which showed that particle size of wheat bran significantly influenced β-galactosidase production. More specifically, the smaller particle size of wheat bran leads to maximum enzyme production (7,610 ± 24 U/gds). The smaller particle size of wheat bran gives a higher surface area which would, allows ease oxygen diffusion, nutrient absorption and assimilation by the mycelia. Hatzinikolaou et al. 2005 has reported that use of wheat bran having smaller particle size for enhanced production of β-galactosidase using *Aspergillus niger.*

### Effect of moisture level and incubation time on β-galactosidase production

**Fig. 3:**
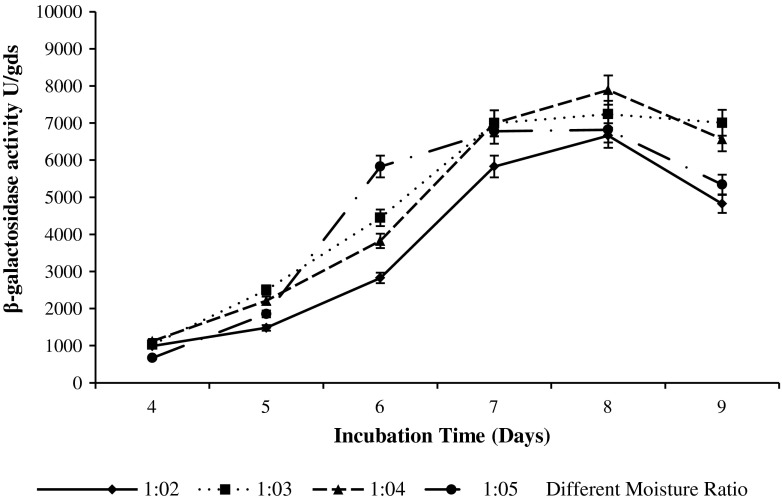
Effect of incubation time and different moisture ratio on β-galactosidase production

β-galactosidase production was investigated (Fig. 3) by adjusting the initial moisture content of SSF from 1:2 to 1:5 (wheat bran: deproteinized acid cheese whey). Increase in moisture level up to 1:4 increases the β-galactosidase production and the maximum β-galactosidase yield (7,890 ± 15.3 U/gds) was obtained at 28 ± 3 °C on eighth day of incubation period then after decrease in the production was recorded. The moisture content in SSF is an important factor that determines the success of the process. Lower than optimal moisture levels may lead to poor solubility of the nutrients into substrate, improper swelling and higher water tension, thereby decreasing product yield (Shah and Madamwar 2005). Akcan (2011) has reported 3 days fermentation period as optimum time for β-galactosidase production whereas, maximum β-galactosidase production was observed after seven days incubation period in case of *A. oryzae* (Nizamuddin et al. 2008).

### Effect of fermentation temperature on β-galactosidase production

**Fig. 4:**
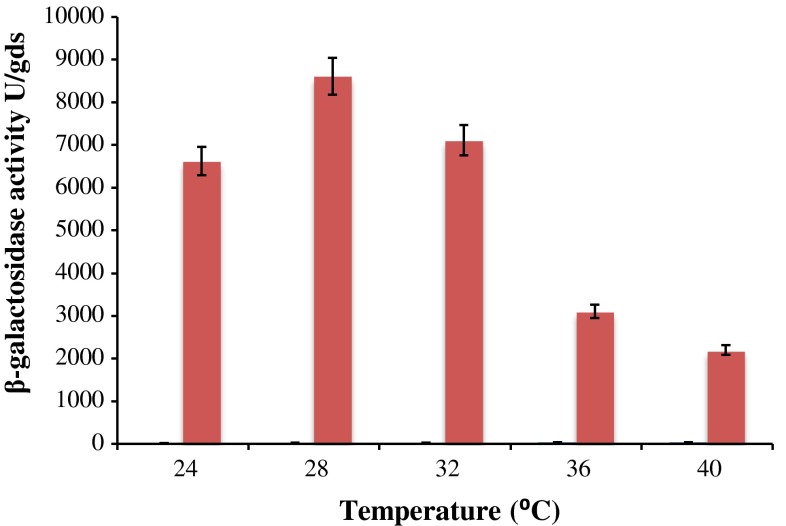
Effect of temperature on β-galactosidase production

To evaluate the optimum temperature for maximum enzyme production, from *A. tubingensis* GR1 fermentation was carried out at different temperatures ranging from 24 to 40 °C with concomitant an increment of 4 °C (Fig. 4). Maximum β-galactosidase production was observed at 28 °C (8,610 ± 19 U/gds) on 8 days of incubation period while enzyme production was comparatively low at 24 and 32 °C. Further increase in the temperature to 40 °C resulted in decrease enzyme production as it did not support the growth of fungus. Awan et al. (2010) has reported maximum galactosidase production in mesophilic range (28–32 °C) using *A. niger* but relatively lower enzyme production was observed at 40 °C.

### Effect of inoculum size on β-galactosidase production

**Fig. 5:**
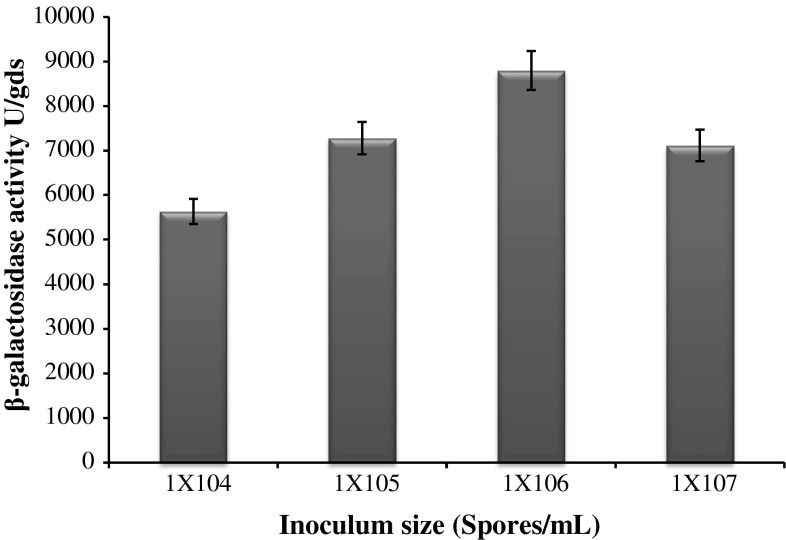
Effect of inoculum size on β-galactosidase production

The effect of inoculum size on the β-galactosidase production based on the number of spores was studied using the spore suspension of 1 × 10^4^, 10^5^, 10^6^ and 10^7^ spores (S)/mL of inoculum (Fig. 5). It was found that the increase in inoculum size resulted in a rapid increase in β-galactosidase production but after 1 × 10^6^ S/mL, a decline in synthesis of enzyme was noticed. This might be due to enhanced competition of cells for carbon, nitrogen and other nutrients, which could lead to scarcity in nutrient availability. This could lead to cessation of growth and reduced enzyme synthesis (Anisha et al. 2008; Awan et al. 2010; Shankar and Mulimani 2010).

### Influence of nitrogen sources on β-galactosidase production

**Fig. 6:**
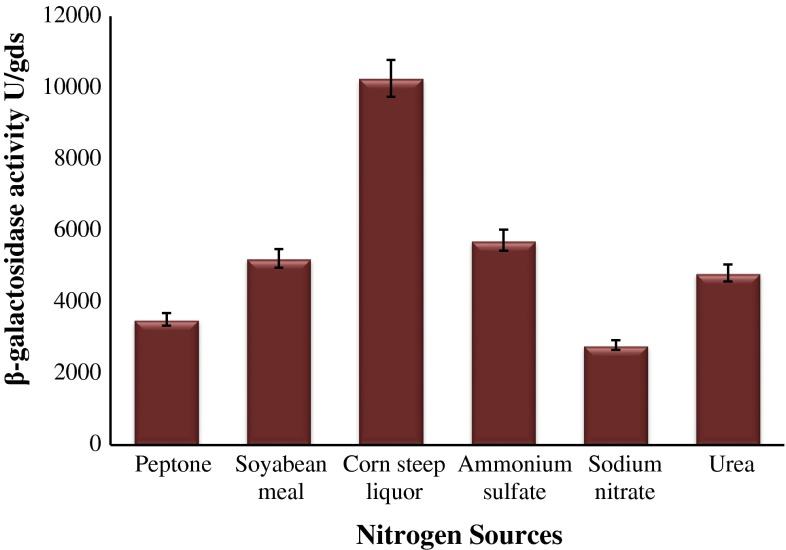
Effect of different nitrogen sources on β-galactosidase production

The effects of organic and inorganic nitrogen sources were studied, keeping the constant concentration (1 g per 5 g of wheat bran) of carbon source. The results were illustrated in Fig. 6. And it was observed that the β-galactosidase production was found to be very from 2,795–10,256 U/gds. Liu et al. (2007) reported that soya bean meal serves as best nitrogen source for β-galactosidase production but in our study we observed that corn steep liquor was found to be most effective supplement for β-galactosidase production (10,256 ± 25 U/gds) from halotolerant *A. tubingensis* GR1. In addition, we observed that supplementation of organic nitrogen sources to the production medium leads to the enhancement of β-galactosidase production. Fekete et al. (2008) reported that metabolic fate of galactose in *A. nidulans* proceeds through reductive pathway and it prefers ammonium salts rather than nitrate as nitrogen source. In our present study, it was observed that the degradation of wheat bran resulted in galactose molecules which serve as carbon source and corn steep liquor supply enough ammonium ions and other vitamins for reductive metabolic fate of galactose.

### Influence of trace element on β-galactosidase production

**Fig. 7:**
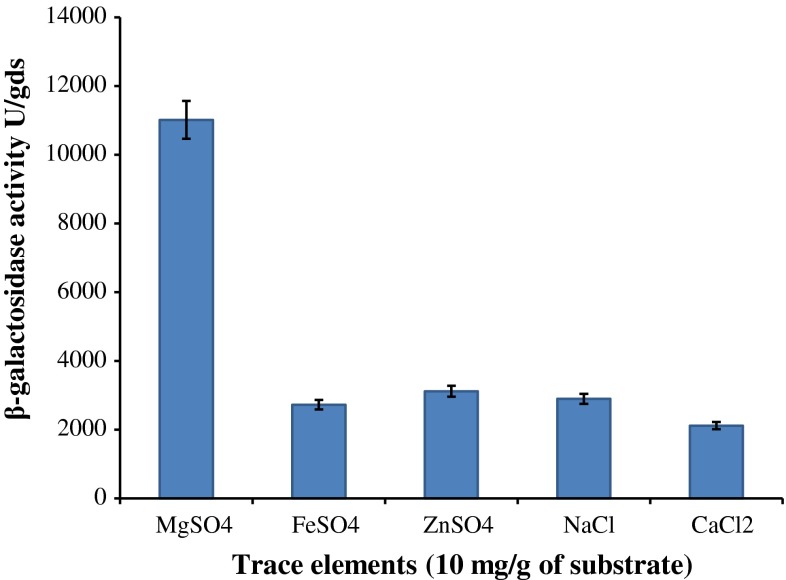
Effect of different trace elements on β-galactosidase production

As shown in Fig. 7, the production of β-galactosidase was enhanced significantly in the presence of 10 mg (per g of wheat bran substrate) of MgSO_4_ (11015.2 ± 12 U/gds) whereas, other trace elements (Zn^2+^, Na^+^, Fe^2+^ and Ca^+2^) did not favor the production of enzyme. Among the different cations, Mg^2+^ ion was found to improve the secretion of β-galactosidase into the medium; this may be attributed to its property of acting as effluxing agent. The involvement of this metal ion in membrane permeabilization and acting as ion channels has been well established (Karpen and Ruiz 2002).

### Response surface methodology (RSM)

The central composite design was employed to evaluate the interaction among the significant factors and also to determine their optimal levels. In the present work, experiments were planned to obtain a quadratic model consisting of 2^3^ trials. The plan included fourteen experiments and two levels of concentration for each factor. In order to study the combined effect of these variables, experiments were performed at different combinations. The central composite experimental plan along with the predicted and observed response for each individual experiment is summarized in Table 2.

The optimum levels of selected variables were obtained by solving the regression equation and by analyzing the response surface contour and surface plots. The larger the magnitude of the *t* value and smaller the *p* value, more significant is the corresponding coefficient (Myers and Montgomery 2002). The regression equation obtained after the analysis of variance (ANOVA) provides an estimate of the level of β-galactosidase production as a function of MgSO_4_ and corn steep liquor concentration.

The production of β-galactosidase may be best predicted by the following model:

2 $$Y = \, 11{,}259 \, + \, \left( {371.70*A} \right) \, + \, \left( {4384.35*B} \right) \, + \, \left( {972*A*B} \right) \, {-} \, \left( {471.06*A^{2} } \right) \, {-} \, \left( {1024.81*B^{2} } \right)$$

where Y = β-galactosidase production (U/gds), A = MgSO_4_ (w/w) and B = Corn steep liquor (v/w).

The statistical significance of the second order model equation was evaluated by *F* test analysis of variance as shown in Table 3 which revealed that this regression is statistically highly significant for β-galactosidase production. The model *F* value 76.15 implies that the model is significant. There is only a 0.01 % chance that a large ‘model *F* value’ could occur due to noise.

**Table 3:** Analysis of variance (ANOVA) for the fitted quadratic polynomial model for level optimization of β-galactosidase production using *A. tubingensis* GR1

Source	Sum of squares	D*f*	Mean square	*F* value	*p* value prob > *F*	
Model	167563059.1	5	33512611.82	76.15	<0.0001	Significant
A-MgSO_4_	1105278.109	1	1105278.109	2.51	0.1517	
B-CSL	153780173.3	1	153780173.3	349.43	<0.0001	Significant
AB	3779136	1	3779136	8.59	0.0190	Significant
A^2^	1638645.26	1	1638645.26	3.72	0.0898	
B^2^	7755623.337	1	7755623.337	17.62	0.0030	Significant
Residual	3520729.258	8	440091.1573			
Lack of fit	2421899.258	3	807299.7527	3.67	0.0976	Not significant
Pure error	1098830	5	219766			
Cor total	171083788.4	13				

*R* ^2^ = 0.96; Adeq Pre = 28.741

A *p* value less than 0.050 indicates that the model terms are significant. Thus in this study, B, AB and B^2^ are significant model terms (Table 3). The lack of fit *F* value of 3.67 implies there is a 9.76 % chance that lack of fit is not significant relative to the pure error. Non-significant lack of fit is good for the model to fit. The *R* ^2^ value (multiple correlation coefficient) closer to 1 denotes better correlation between observed and predicted values. The coefficient of variation (CV) indicates the degree of precision with which the experiment is compared. The lower reliability of the experiment is usually indicated by high value of CV. In the present case, low CV (6.37) denotes that the experiment performed is reliable. Adequate precision measures the signal-to-noise ratio. A ratio greater than 4.00 is desirable. In present study, the ratio is 28.47 which indicates an adequate signal. This model can be used to navigate the design space.

**Fig. 8:**
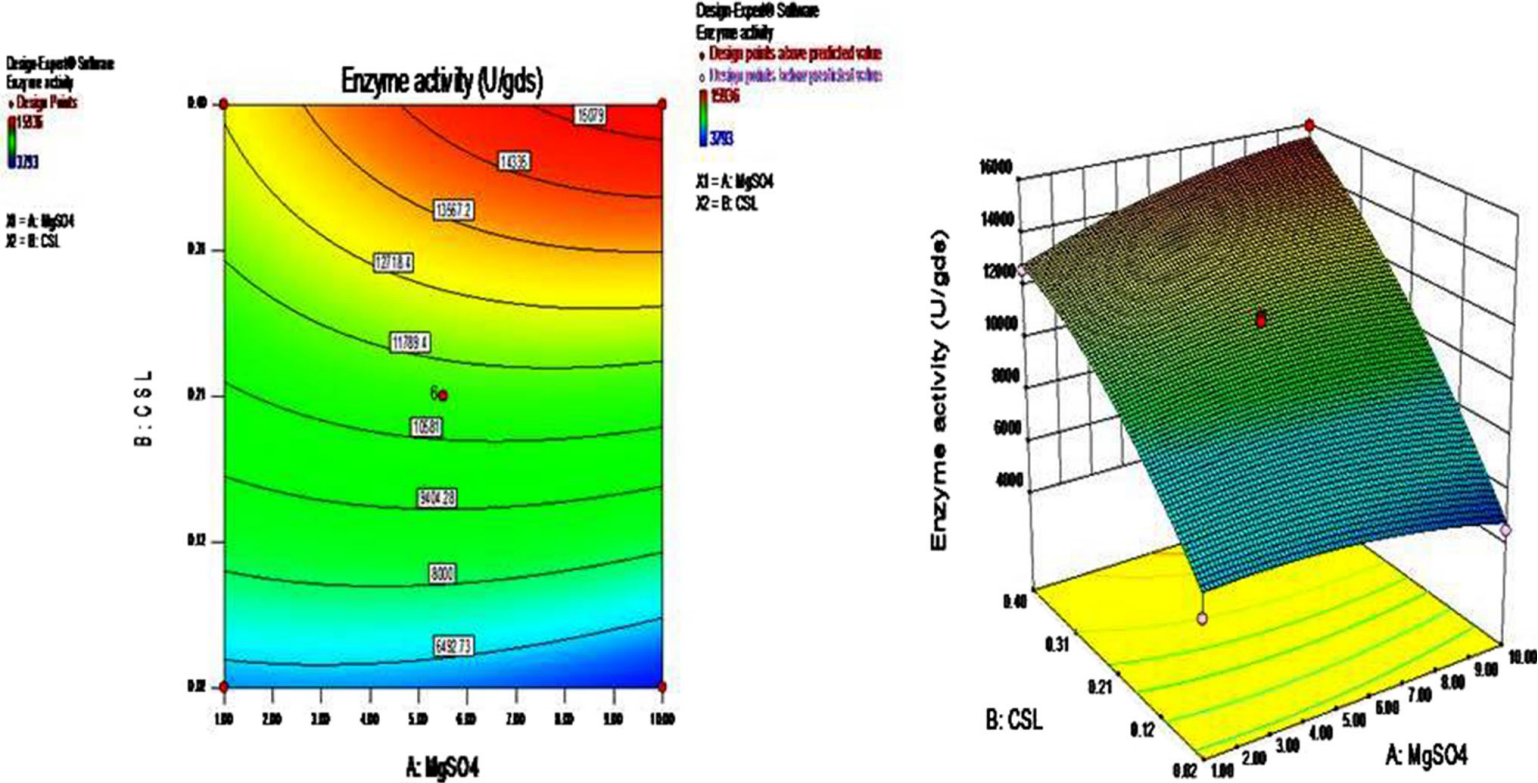
Response surface graph showing interaction effect of MgSO_4_ and corn steep liquor on β-galactosidase production

The interaction effect of variables on β-galactosidase yield was studied by changing the levels of any one independent variable while keeping the second independent variable at its constant level. The response surface plots or contour plots can be used to predict the optimal values for different test variables. Therefore, a single response surface plot was obtained by considering the possible combinations. As shown in Fig. 8, three-dimensional response plot describes the behavior of β-galactosidase production, main effect, interaction effect, and squared effect (nonlinear) of MgSO_4_ and corn steep liquor at different concentrations. The interaction of both the components was found to be positive and showed significant effect on the β-galactosidase production. Increase or decrease in the MgSO_4_ level alone did not show any prominent effect on the enzyme production, but increase in the corn steep liquor level leads to the gradual increase in the enzyme production. Both the components at their higher concentration in the production media showed significant effect on β-galactosidase production. According to the response surface point prediction analysis, MgSO_4_ concentration of 10 mg/g (w/w) and corn steep liquor 0.4 mL/g (v/w) leads to enhance β-galactosidase yield up to 15,936 U/gds.

### Validation of the quadratic model

In order to confirm the above-mentioned optimized medium constitution and condition an experiment for β-galactosidase production was performed in triplicate. Under these suggested conditions the mean value of the β-galactosidase yield was found to be 15,936 U/gds, which was higher than the predicted value of 15491.17 U/gds. Thus, the model developed was accurate and reliable for predicting the production of β-galactosidase by halotolerant *A.* *tubingensis* GR1.

## Conclusions

In this study, the improvement of β-galactosidase production by halotolerant *A.* *tubingensis* GR1 using agro-industrial wastes under SSF has been reported. The individual and interactive role of nitrogen source and magnesium sulphate concentration on β-galactosidase yield was investigated by Response Surface Central Composite Design. To the best of our knowledge, it is for the first time that statistical approach has been employed and showed significant results for optimizing the medium components for maximal β-galactosidase production under solid state fermentation using a halotolerant *Aspergillus* spp. The enzyme yield and the production were found to be significantly influenced by corn steep liquor and magnesium sulphate along with wheat bran and deproteinized acid cheese whey. The data obtained after optimization has resulted in 15,936 U/gds compared to unoptimized medium (5661.4 U/gds) for enzyme production.
